# Consultations in the Country

**Published:** 1874-06

**Authors:** 


*THE MEDICAL RECORD—April*, 1874:

Consultations in the Country.—Notwithstanding there are a great many rules laid down in the code for the government of consultations, there are still some points in the unwritten law of medicine in regard to them which are either not understood or are purposely ignored by very many practitioners. For a thoughtless or ill-disposed person there is always an opportunity, which, if ignorantly or meanly seized during a consultation, can be made to be injurious, nay, ruinous, to the regular attendant. The dangers are indeed so great that, in order to guard against them, the consulting gentleman has his actions hemmed in by all sorts of restrictions, and is always expected to strain a point and conscientiously use every honorable means to strengthen and maintain that confidence and esteem which should always exist between the family physician and patient. There are different ways of doing this, which must be modified by circumstances, surroundings and conditions.

The very office of consulting physician generally carries with it such advantages that almost always the claim for protection and support must come from the gentleman in direct charge of the case. The necessity for a consultation generally proves the existence of a desperate case—one in which extra counsel is required. The consulting physician is either chosen by the patient, the physician, or both; and for the time being is looked upon, by the friends of the patient at least, as an adviser whose opinions concerning the particular case in question are of a peculiar value. We maintain that the consulting physician, while he admits to himself the necessity for his services, and accepts the choice as a compliment to his skill and abilities, should be exceedingly particular to discharge his delicate and responsible duties to the satisfaction of the patient's friends, and especially to the patient's physician.

Consultations are always useful, and all other things being equal, cannot be held too frequently. They are not only a great relief to the minds of our patients and their friends, not only a source of satisfaction to ourselves, but in the main tend to establish a confidence in the healing art, a respect for medicine as a science, a more thorough appreciation of its resources, and a conviction in the minds of our patients that we, as a class of professional men, are anxious and willing to do everything which a combination of skill and experience can effect. For either party to the consultation to attempt to make capital of the circumstance, is not only unprofessional, but unbecoming that spirit which seeks the welfare of suffering humanity in the light of a strict and conscientious interpretation of scientific truths. Under these circumstances, individual considerations and self-glorifications should be lost in the desire to elevate the profession as a whole. We do not believe that anything tends to destroy this confidence more effectually than an attempt on the part of either member of the consultation to prove to non-professionals the possession of a superior knowledge and skill.

Despite all our efforts, there must be some wrongs inflicted, some misunderstandings initiated, some unwarrantable estimates of relative skill made by our patrons. All cannot be expected to be equally informed, skilful or discriminative; we all differ in these respects, one with another. It, however, should always be the endeavor of the new comer into a case, the one whose aid and counsel is sought, to reduce the chances of an over-estimate of his abilities to the minimum. Taking a strictly honorable view of such relations as should exist between the consulting and family practitioner, this should always be the case. A consulting physician or surgeon is called in to aid both the family attendant and the patient, and nothing more. He does not sacrifice any of his interests by a manly course of action to both parties, and as his attendance with a given case is expected to cease with the consultation, there is no temptation for him to aspire for any further or future control of the patient; at least there should be none.

Cases not unfrequently arise in which peculiar circumstances should alter the usual relations of practitioners to each other; for instance, where special operations are to be performed, in which a special experience is required. The one who has such skill should give the patient the benefit of it, and no considerations of professional pride, and no illiberal interpretation of the code should interfere. It would be worse than criminal for any practitioner merely bent on maintaining a hold upon his patient to insist upon performing an operation which at best he could do in a very bungling manner, in the presence of the other party to the consultation, who may be an expert. But in these cases common courtesy generally adjusts all such difficulties, and when the professional positions of each are honorably and properly balanced, no advantage can be taken by one over the other.

